# Cloning, and Molecular Characterization of Polymorphic Iranian Isolate *Theileria annulata* Surface Protein (Tasp)

**Published:** 2012

**Authors:** N Sadr-Shirazi, P Shayan, B Eckert, E Ebrahimzadeh, N Amininia

**Affiliations:** 1 Department of Parasitology, Iranian Center for Ticks and Tick-borne Diseases, Faculty of Veterinary Medicine, University of Tehran, Tehran, Iran; 2 Investigating Institute of Molecular Biological System Transfer, Tehran, Iran

**Keywords:** *Theileria annulata*, DNA, mRNA, SMART-cDNA, Cloning, *TaSp* protein, Iran

## Abstract

**Background:**

Because of the strong immunologic responses of surface protein *TaSp* in *Theileria annulata* infected host, we tried to characterize this protein in a *T*. *annulata* isolate from Iran.

**Methods:**

The RNA prepared from *T. annulata* infected cells was used to produce SMART-DS-cDNA. The Double strand cDNA was then amplified with primers derived from *TaSp* mRNA sequences. The PCR product was cloned in pTZ57R/T vector, sequenced and registered under accession no. **JQ003240** in GenBank.

**Results:**

The sequence analysis showed 90%–94% nucleotide sequence identity and 68%–94% amino acid homology to the corresponding sequences of *TaSp* gene by *T. annulata*, *T. sp. china I*, *T. sp. china* and *T. lestoquardi* and three *T. annulata* reported from Iran respectively. Interestingly, the sequence analysis also showed small nucleotide sequence region near the 5‘ end in which the presented *TaSp* protein differed very strongly from the other known *TaSp* sequences. For the preparation of the recombinant protein, the cDNA was cloned in pQE-32 vector, the recombinant protein was prepared and assayed by *Theileria* infected bovine serum.

**Conclusion:**

The polymorphism in *TaSp* gene could be detected in intra- as well as inter species. The different characterized *TaSp* proteins had a common identic region, which may be helpful for development of broad band vaccine based on the recombinant proteins. The polymorphism in this gene, make this protein also interesting for the diagnostic purposes.

## Introduction

Tropical or Mediterranean Theileriosis caused by the protozoan parasite *Theileria annulata*, is distributed in the middle east and regions of southern Europe, North Africa, India and China (1). *Theileria* genus differentiates themselves from the *Babesia* parasites by having a schizont stage in their life cycle, which parasitized the host's white blood cells (2). Ticks of the genus *Hyalomma* are the common vectors of this protozoan parasite that transmit the parasite to the final hosts including cattle and water Buffalo (3).

Diagnosis of disease can be performed by demonstrating of the annular forms of piroplasms in the erythrocytes during the acute phase and especially by detection of schizonts in the white blood cells using Giemsa staining method. This method belongs to the most used diagnostic methods in Iran. Since the Giemsa staining method is unspecific and can be accompanied with some technical problems and in some cases needs special experiences especially by low parasitaemia, can not be used as a reliable method for diagnosis of theileriosis (4). PCR is useful method for the detection of *T. annulata* agent in carrier cattle even where parasitaemia may be low (5). It is also the most specific method for differential diagnosis of piroplasms using different sources of blood samples, including already stained blood smears (6). The only disadvantage of this method is that, the PCR technique can not differentiate between the acute phase, latency period or carrier animals. The indirect fluorescence antibody test (IFAT) had more sensitivity than the Giemsa staining method for detection of *T. annulata* in cattle (7). One of the disadvantages of the diagnostic methods like IFAT based on the polyclonal antibody against the whole antigens prepared from *Theileria* agent is the cross-reactivity among the *Theileria* species, which was described long time ago by Burridge et al. (8). Production of recombinant proteins in recent years led to the development of new ELISA for detection of several important *Theileria* species such as *T. parva* and *T. annulata* (9, 10).

The characterization of *T. annulata* surface protein *TaSp* and its followed application in indirect ELISA makes *TaSp* protein as a serious candidate molecule for developing of detecting and/or controlling tools (11). Application of *T. annulata* surface protein in an indirect *TaSp-*ELISA in Sudan showed a sensitivity of 99.1% and specificity of 90.47% compared to the IFAT test which were used as a reference test there (12). *TaSp* is a membrane protein that contains three membrane domains, with a large segment containing a polymorphic region facing the cytoplasm of the host cell (11). Since the tropical theileriosis caused annually high economical losses in Iran, it is important to perform epidemiological studies dealing with the prevalence of infection, needs a reliable diagnostic tool for screening of sera.

The aim of this study was to characterize the *TaSp* protein in the *T. annulata* isolate from Iran and compare it with the known corresponding sequences registered in GenBank.

## Materials and Methods

### DNA Extraction and PCR

Total DNA was extracted from a jugular lymph node biopsy of a *Theileria* infected female dairy cow using DNA Extraction kit (MBST, Tehran, Iran), according to the manufacturer protocol. Two pair of primers was designed for amplifying the bovine β-actin and *T. annulata* 18s rRNA encoding genes. In two separate PCR program, approximately 10 ng DNA solution was used for the PCR analysis that performed in 100 µl total volume including 10x PCR buffer, 2.5 U Taq polymerase (*Cinnagen*, Iran), 2 µl of each primer (20 µM, *Cinnagen*, Iran), 2 µl of each dATP, dTTP, dCTP and dGTP, (200 µM, *Fermentas*), 1.5 mM MgCl_2_, in automated Thermo cycler (*MWG Biotech* Primus, Germany) with the following program: 5 min incubation at 95°C to denature double strand DNA, followed by 38 cycles of 45 s at 94 °C (denaturation), 45 s at 55 or 60 °C (annealing) and 45 s at 72 °C (extension) and an additional extension step at 72 °C for 5 min. Annealing temperature used for β-actin and *T.annulata* primer sets were 60 °C and 55 °C, respectively. The used primers were listed in Table I. The expected PCR product length for β-actin and *T. annulata* were 639 and 430 bp, respectively. 10 µl of each PCR product were subjected to electrophoresis on a 1.5% agarose gel in TBE buffer and visualized under UV light by ethidium bromide staining.

**Table 1:** The nucleotide sequences of the primers used in this study

No	Name of primer	Accession no.	Nucleotide sequences	PCR Product Length (bp)
1	Bovine β-actin sense	**NM173979**	5' atcactgccctggcacccag 3'	639
2	Bovine β-actin antisense	**NM173979**	5'cttagagagaagcggggtggc3'	
3	*T. annulata* sense	**AY150056.3**	5'cacagggaggtagtgacaag3'	430
4	*T. annulata* antisense	**AY150056.3**	5'ctaagaatttcacctctgacag3'	
5	*Tasp* sense	**AJ316249.1**	5' atcggatcccctatcgattttgatcccaatgatg 3'	704
6	*Tasp* antisense	**AJ316249.1**	5' atcaagctttcagtccaatgcataagcacag 3'	
7	Nested *Tasp* sense	**AJ316249.1**	5‘cagcctttggaccctaatcaa3‘	600
8	Nested *Tasp* antisense	**AJ316249.1**	5‘ctgtaggccttcaaacatgga 3‘	
9	Vector pTZ57R/T- sequencing sense	Partial nucleotide sequence map from Fermentas company	5‘gaattcgagctcggtacctc3‘	116
10	Vector pTZ57R/T- sequencing antisense	Partial nucleotide sequence map from Fermentas company	5‘accatgattacgccaagctc3‘	

### SMART-Double Strand (DS)-cDNA synthesis

Total RNA was extracted immediately from the second biopsy from the same lymph node described in section 2.1., using TriPure Reagent (Roche, Basel, Germany) according to the manufacturer instruction. One microgram of total RNA was subjected to SMART cDNA synthesis following manufacturer's instruction (Clontech, *USA*). Ten micro liter of SMART cDNA was amplified with the primer supplied by Kit (Clontech, *USA*) under following conditions: Denaturation 1 min at 95 °C followed by 27 amplification cycles of 15 s at 95 °C (denaturation), 30 s at 65 °C (annealing), 6 min at 68 °C (extension). Finally, the PCR products were analyzed by agarose gel electrophoresis. Since small amount of biological materials was available, we used SMART technology in which a customized oligo (dT) primer was used to selectively prime full length mRNAs for preparation of representative cDNA probe for all mRNAs in total RNA. The cDNA can then be amplified several times with the customized primers to supply a reservoir in desired amounts for future studies.

### Amplification of TaSp gene

One µl of SMART-DS-cDNA was amplified using specific primers derived from gene coding for *TaSp* protein (Table I) under following conditions: Initial denaturing temperature at 95 °C for 5 min, 38 cycles of 95 °C for 45 sec (denaturation), at 68 °C for 45 sec (annealing), at 72 °C for 45 sec (elongation) and an additional elongation for 10 min at 72 °C. The sense primer had at the 5‘-end the nucleotide sequences GGATCC which is recognition site for restriction endonucleasis Bam HI and the antisense primer had at the 5‘-end the nucleotide sequences AAGCTT which is recognition site for restriction endonucleasis HindIII. Expected PCR product was 704 bp in length. 100µl of PCR product was purified with PCR Product Purification Kit (MBST, Tehran, Iran) as manufacturer's protocol.

### Nested-PCR of TaSp encoding gene

In order to confirm that the *TaSp* PCR product, was *TaSp* specific, two inner primer set were designed (Table I). The Nested PCR was performed on 10 ng of purified *TaSp* PCR product as nearly the same program for *TaSp* PCR with the distinct annealing temperature at 62°C. The expected nested PCR product was 600 bp in length. The nested PCR product was subjected to electrophoresis on 1.5% agarose gel and visualized under UV light by ethidium bromide staining.

### Cloning in pTZ57R/T vector

One hundred µl of *TaSp* PCR product was purified using PCR product kit (MBST, Tehran, Iran) according to the manufacturer protocol. Four µl of the purified PCR product was added to the solution containing 3 µl of plasmid vector pTZ57R/T DNA ( 0.18 pmol ends), 3µl of 5X ligation buffer, 1µl T4 DNA Ligase and 19 µl de ionized water, then incubated at 22°C for 1 hour.

### Preparation of competent cells and Transfection

*E. coli* DH5-α strain was used for preparing of competent cell and further transformation processes (13).

### Nucleotide sequence analysis

The recombinant plasmid containing *TaSp* was extracted in large quantity using the Plasmid Extraction Kit (MBST, Tehran, Iran) according to the manufacturer protocol. The extracted plasmid was first analyzed on agarose gel under UV condition and sequenced according to the Sanger Method by Kowsar Company (Tehran, Iran).

### Cloning in pQE-32 vector

The recombinant *TaSp*-pTZ57R/T-vector was digested with BamHI (Fermentas). The purified Bam HI-digested plasmid DNA was then treated with the restriction endonuclease Hind III enzyme (Fermentas). The cut 704 bp insert DNA was then extracted from the agarose gel using DNA Extraction Kit from Agarose Gel (MBST, Tehran, Iran) according to the manufacturer protocol. The pQE-32 plasmid DNA was double digested with the same restriction endonucleases as described above. The *TaSp*-DS-cDNA product was cloned in the multicloning site (BamHI and HindIII site) of pQE-32 plasmid using rapid DNA ligation kit (Roche, Germany). The recombinant *TaSp*-pQE-32 was then transferred to the competent bacteria DH5-α and analyzed by agarose gel and sequencing. The recombinant *TaSp* protein was finally analyzed by SDS-PAGE and western blotting.

### SDS-PAGE and Western blot

The overnight-grown transfected *E. coli* was incubated in LB medium containing ampicillin until OD650 of 0.6–0.8 was achieved. Subsequently, IPTG was added to the culture and incubated under shaking condition. The cultured cells were centrifuged and the cells in pellet were lysed. The lysed cells were centrifuged and supernatant was collected for SDS-PAGE and western blot analysis. The recombinant *TaSp* protein was additionally extracted using Ni-NTA Spin Kit (Qiagen, Hilden, Germany), according to the manufacturer's instruction. Extracted *TaSp* recombinant protein was separated on 12% SDS-PAGE. The protein bands were either stained with Coomassie brilliant blue or transferred to the nitrocellulose membrane using Semi-Dry trans-Blot (BioRad, USA). For western blot analysis the free binding sites on the membrane were first blocked with 3% bovine serum. Subsequently, the membrane was incubated in a positive serum (1:500 dilution) obtained from *T. annulata* infected calf. Horseradish-conjugated rabbit anti-bovine Ig (Dako, Denmark) (1:2,000) were added to the washed membrane and incubated for 1 h at RT. The positive reaction was developed using DAB (Sigma, USA) as substrate under visual observation within 5 min. In all experiments, samples of *E. coli* containing not inserted plasmid and *E. coli* with no plasmids extracted proteins were used as negative controls. Serum dilution (1:500) was then selected for the test sera.

## Results

**Fig. 1:**
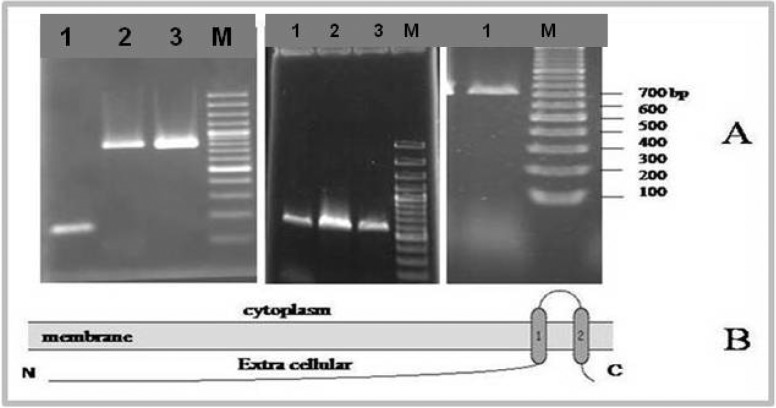
A) The SMART-DS-cDNA was amplified using specific primers for TaSp gene (Lane 1 at the right side). The specificity of the amplified RT-PCR products were confirmed by nested PCR (Lane 1, 2 and 3 at the middle side). The Amplicon was cloned into the vector pTZ57R/*T* and analyzed using primers derived from nucleotide sequences flanked the cloned insert. Lane 2 and 3 at the left side were the PCR products from recombinant vector and lane 1 was the amplicon of vector without insert. M was 100 bp DNA marker. B) The transmembrane helices structure analysis of presented *TaSp* protein from *Theileria annulata* isolated from Iran using Toppred program.

To analyze the *TaSp* protein in the presented *T. annulata* isolate from Karadj province in Iran, lymph node biopsy material from naturally infected cattle with *T. annulata* was used. To show that the cattle harbored *Theileria* agent, the DNA was isolated from the biopsy. The second biopsy was used for RNA extraction. PCR analysis of the extracted DNA with primer pair derived from bovine *β-actin* gene showed that the extracted DNA was suitable for amplifying by PCR (data not shown). The DNA was subsequently amplified using primer pair derived from *T. annulata* 18S rRNA gene to detect *Theileria* agent in lymph node (data not shown). Since a small amount of biological material was available, the SMART technology was used for amplifying of *TaSp* specific cDNA. For this aim, the RNA was first extracted and used for generating of SMART-cDNA. SMART-cDNA was first amplified using primer supplied by Clontech Company. The resulting double strand cDNA was then used as cDNA sample for amplification of *TaSp* mRNA by RT-PCR using *TaSp* specific primer pair. Figure 1A (at the right side) showed an expected RT-PCR product of approximately 704 bp in length. The nested PCR analysis showed that the above mentioned PCR product was *TaSp* specific (Fig. 1A, at the middle side). To determine the nucleotide sequence of the *TaSp* amplicon, the PCR product was cloned into the pTZ57R/*T* vector. After cloning procedure colony PCR was done using primers specific for amplifying the part of pTZ57R/T vector flanked the *TaSp* inserted region. Lanes 2 and 3 of Fig. 1A (at the left side) showed PCR amplicon of 820 bp *TaSp* cloned region in the vector and lane 3 showed PCR amplicon of a negative clone.

The insert in recombinant plasmid was subsequently sequenced. The partial nucleotide sequence including corresponding amino acid sequence of the *TaSp* gene in *T. anuulata* isolated from Iran was registered under accession no. **JQ003240** in GenBank. The comparison of the nucleotide sequence showed identity of 94%, 94%, 92% and 90% homology to the nucleotide sequences of *TaSp* genes by *T. annulata*, *T sp. china I*, *T sp. China* and *T. lestoquardi*, previously registered in GenBank under accession nos. **AJ316249.1**, **AY274329.1**, **DQ120058.1**, and **AY274335.1**, respectively.

*TaSp* protein gene was also previously partially determined in 3 different *T. annulata* isolates from Iran. Two of these isolates were collected from the nearly same region (Boin-Zahra) whereas the other one was isolated from Karaj region. The nucleotide sequence analysis showed that the available *TaSp* gene sequence from 2 isolates from Boin Zahra (**EF092921.1** and **EF092922.1**) had 99% homology to each other, whereas these sequences had 94% homology to the isolate from Karaj (**EF092920.1**). Interestingly, the presented *TaSp* nucleotide sequence had only 92% homology to the corresponding sequence from Karaj, 93% homology to the sequence registered **EF092922.1** and 94% homology to the other sequence from Boin Zahra (**EF092921.1**).

**Fig. 2:**
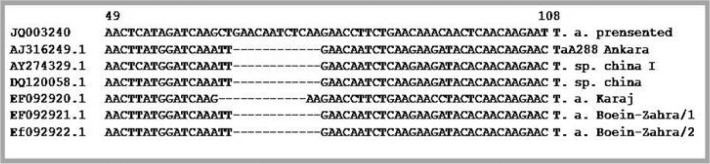
*Comparison of nucleotide sequence of TaSp protein gene in presented isolate under* accession number. **JQ003240** with the corresponding sequences in *T. annulata (Ankara), T. sp. china I, T. sp. China, T. annulata* (Karaj), *T. annulata* (Boein-Zahra/1) and *T. annulata* (Boein-Zahra/2) registered under accession no. **AJ316249.1, AY274329.1, DQ120058.1, EF092920.1, EF092921.1** and **Ef092922.1** in GenBank respectively. In the presented *TaSp* nucleotide sequence (JQ003240) there are twelve nucleotides from position 66 to 78 which only exist in presented *TaSp* sequence and could be determined as the hypervariable region in the polymorphic part of the *TaSp* gene

Interestingly, there was 12 nucleotides occurring at the 5‘ region of *TaSp* gene from *T. annulata* presented in this paper, which was absent in the other examined *TaSp* gene genes (Fig. 2).

**Fig. 3:**
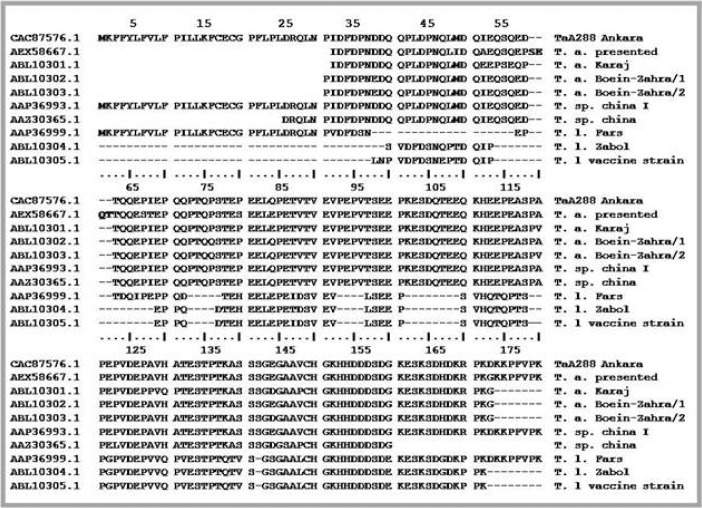
The amino acid sequence of the presented *TaSp* protein from *T. annulata* isolated from Iran (**AEX58667.1**) was compared with the corresponding sequences in *T. annulata* (Ankara), *T. annulata* (Karaj), *T. annulata* (Boein-Zahra/1), *T. annulata* (Boein-Zahra/2), *T sp. china I*, *T sp. China, T. lestoquardi* (Fars), *T. lestoquardi* (Zabol) and *T. lestoquardi* (vaccine strain) registered under accession no. **CAC87576.1**, **ABL10301.1, ABL10302.1, ABL10303.1, AAP36993.1, AAZ30365.1, AAP36999.1**, **ABL10304.1** and **ABL10305.1** in GenBank respectively. There are four amino acids from codon 59-61 (SEQT) which only exist in the presented *TaSp* amino acid sequence and could be considered as the hypervariable region in the polymorphic region of *TaSp* protein

The presented *TaSp* product coded from codon 31 to codon 265 (end codon) of the proteins registered in GenBank. The first 30 absent codons contained signal peptide (from codon 1 to codon 18) and first transmembrane region (from codon 19 to codon 30) of the protein. The structure analysis using TMHMM server v. 2.0 programs, which analyzed the prediction of transmembrane helices in proteins, showed that the amino acid sequences from codon 1 to codon 176 of presented recombinant protein should be located in the extracellular region of the parasite. The amino acid sequences from codon 177 to codon 179 should be participated in conformation of the second transmembrane helix and the sequences from codon 200 to codon 210 should form the inside loop region. The third transmembrane helix predicted to be located from codon 211 to codon 233 followed by the codon 234 that conformed the outside part. (Fig. 1B). The schematic representation of *TaSp* protein embedded in the membrane was predicted using Toppred program (Fig. 1B) The presented recombinant *TaSp* protein also comprised of about 41.4% neutral (D, E, G, Q, S, T), 25.2% hydrophobic (A, Y, C), 21.3% very hydrophobic (F, L, I, M, V, W) and 11.9% hydrophilic amino acid residues (H, K, N, P, R), (Fig. 3).

**Fig. 4:**
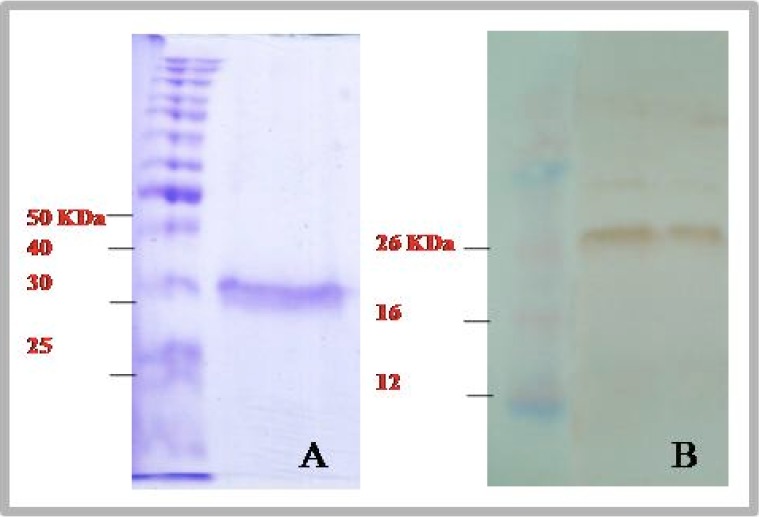
The recombinant *TaSp* protein from *T. annulata* isolated from Iran was prepared, purified by Ni-NTA Spin Kit pQE-32 and analyzed by SDS-PAGE (A) and by western blotting using a serum collected from a *T. annulata* infected cattle (B)

The amino acid sequence analysis showed 94%, 94%, 88% and 68% homology to the corresponding amino acid sequence of *T. annulata*, *T sp. china I*, *T sp. China* and *T. lestoquardi* registered under accession no.**CAC87576.1, AAP36993.1, AAZ30365.1** and **AAP36999.1** in GenBank respectively (Fig. 3). Also, the amino acid sequence analysis showed 91%, 89% and 89% homology to the corresponding *TaSp* amino acid sequence of isolates from Iran namely *T. annulata* isolate Boein-Zahra/2, *T. annulata* isolate Boein-Zahra/1 and *T. annulata* isolate Karaj registered under accession nos. **EF092922.1**, **EF092921.1** and **EF092920.1**, respectively. Interestingly, the presented *TaSp* amino acid sequence had four additional amino acids located in positions from codon 59 to codon 62 (SEQT), which were not presented in other *TaSp* sequences registered in GenBank. The comparison of amino acid sequences of presented *TaSp* protein with the corresponding sequences in *T. lestoquardi* showed that these two sequences had the main differences in N-terminus region and high differences were seen up to codon 120. The conserved sequences from codon 143 to the end of C-terminus were identical between the two mentioned species. The *TaSp*-SMART-cDNA was cloned into the expression vector pQE-32 and the prepared recombinant protein was then analyzed by western blotting using a serum collected from a *T. annulata* infected cattle. The analysis showed that the recombinant *TaSp* protein could be detected by the above mentioned serum (Fig. 4).

## Discussion

Tropical theileriosis caused by the protozoan parasite *T. annulata*, is distributed in the Middle East and regions of southern Europe, North Africa, India and China (1). The genus of *Hyalomma* ticks as the main vector for *T. annulata* were reported from tropical and subtropical as the most geographical regions of Iran (14, 15), have the potential conditions for occurrence of tropical Theileriosis. Theileriosis caused annually high economical losses worldwide and therefore the control and prevention of this disease is very important in animal health management. Many epi-demiological studies were performed using molecular techniques such as PCR, nested-PCR, PCR-RFLP or reverse line blot (RLB) (16–18). At the immunogenic protein level, Shayan et al. (19) could characterize a partial sequence of a cDNA coding for a protein expressed by *T. annulata* schizonts, which was later completely sequenced by Schnittger *et al*. (20) and was shown to be a surface protein named *TaSp*. It was shown that *TaSp* occurs as a single-copy gene and is expressed in the sporozoite and schizont stages of the parasite (20). Homologues of this gene were also shown to exist in other *Theileria* species namely, *T sp. china I*, *T sp. China* and *T. lestoquardi* registered in GenBank under accession nos. **AY274329.1**, **DQ120058.1** and **AY274335.1** respectively. The recombinant *TaSp* protein was first prepared in the Jabbar Ahmed group in Research Center Borstel (Germany) and used as a screening tool by indirect ELISA (11, 21). They have applied also this recombinant protein successfully for sera screening in epidemiological surveys in Sudan (12).

The followed investigations showed that *TaSp* protein is the most immunogenic protein of *T. annulata* among the other characterized and prepared recombinant proteins such as *TaD* (22), *TaSE* (23) and *TamtHSP70* (24). Validation of *TaSp* protein for detection of *T. annulata* infection in indirect ELISA was confirmed by Seitzer (25). The analysis of gene encoding *TaSp* molecule of *T. annulata* isolate presented here showed 94%, 94%, 92% and 90% homology to the nucleotide sequence of the *TaSp* gene by *T. annulata*, *T sp. china I*, *T sp. china* and *T. lestoquardi* documented under accession nos. **AJ316249.1**, **AY274329.1**, **DQ120058.1** and **AY274335.1** respectively. The presented sequence had also 94%, 93% and 92% homology to the corresponding sequences determined by *T. annulata* isolates reported from Iran registered in GenBank under accession numbers **EF092921.1**, **EF092922.1** and **EF092920.1** respectively. It is noticeable that there were 12 nucleotides (from nucleotide position 66 to position 78) in the presented *TaSp* protein encoding gene which were absent in other *T. annulata TaSp* sequences mentioned above. The amino acid sequence analysis showed 94%, 94%, 88%, 68%, 91%, 89% and 89% homology to the corresponding amino acid sequence of *T. annulata*, *T sp. china I*, *T sp. china*, *T. lestoquardi,T. annulata* isolate Boein-Zahra/2, *T. annulata* isolate Boein-Zahra/1 and *T. annulata* isolate Karaj registered under accession nos. **CAC87576.1**, **AAP36993.1**, **AAZ30365.1**, **AAP36999.1**, **ABL10303.1**, **ABL10302.1** and **ABL10301.1** registered in GenBank respectively. The minimum similarity is between the *T. annulata* SP of our isolate and *T. lestoquardi* (68%) and surprisingly, the maximum identity are between our *TaSP* amino acid sequence and two *Theileria sp*. from China. A noticeable difference between the above mentioned sequences was the presence of a region consisting of four amino acids (SEQT) in the presented *TaSp* protein which was absent in the other documented genes also in corresponding genes documented from Iran. The extreme polymorphism in this region between different *TaSp* genes may show that this region most probably is not involved in the molecular interaction with other cytoplasmic proteins of the host cell. The most frequent amino acid of the presented *TaSp* protein using ProtParam online program was Glutamic acid (11.1%), which was as the same as three other sequences of *T. annulata* isolate Boein-Zahra/1 (17%), *T. annulata* isolate Boein-Zahra/2 (16.5%) and *T. annulata* isolate Karaj (17.4%). Glutamic acid was also the most amino acid in T. sp. china (16%). In contrast to these isolates reported from Iran, the most frequent amino acid of *T. annulata* (GenBank accession number. **CAC87576.1**), *T sp. China 1*, *T. lestoquardi* and *T. lestoquardi* Zabol isolate were Leucin and Prolin (9.3%), Leucin (9.6%), Leucin (11%) and Prolin (12%), respectively. In spite of the differences in the amino acid sequences between the *TaSp* proteins in the mentioned *Theielria* species, no significant differences could be shown in the predicted third structure of the proteins. In contrast to the amino acid region at the C-terminus (the last 114 amino acids), a high differences could be detected at the N-terminus between the amino acid sequence of *TaSp* proteins of *T. lestoquardi* and *Tasp* protein in *T. annulata* including the isolates from Iran and *T. sp. china* I. Since from one side the antibody reactivity to the recombinant *TaSp* protein was observed in sheep infected with *T. lestoquardi* and *T. sp*. (China) (26, 27) and from the other side *T. annulata* can infect sheep (28, 29), the mentioned sequence difference could be used for the establishment of a specific detection tool for *T. lestoquardi* infections.

In conclusion, the presented study showed the presence of polymorphism in the *TaSp* gene from *Theileria spp*. This polymorphism could be also detected intra- as well as inter species. The different characterized *TaSp* proteins had a common identic region, which may be helpful for development of broad band vaccine based on the recombinant proteins. The polymorphism in this gene, make this protein also interesting for the diagnostic purposes. The antibody against this protein can be helpful for studies dealing with the molecular interaction between this protein and the host proteins in macroschizont harboring cells.
